# miSolRNA: A tomato micro RNA relational database

**DOI:** 10.1186/1471-2229-10-240

**Published:** 2010-11-08

**Authors:** Ariel A Bazzini, Ramón Asís, Virginia González, Sebastián Bassi, Mariana Conte, Marcelo Soria, Alisdair R Fernie, Sebastián Asurmendi, Fernando Carrari

**Affiliations:** 1 Instituto de Biotecnología, Instituto Nacional de Tecnología Agropecuaria (IB-INTA) (Partner group of Institution 5), P.O. BOX 25, B1712WAA Castelar, Argentina; 2 CIBICI, Facultad de Ciencias Químicas Universidad Nacional de Córdoba, CC 5000, Haya de la Torre y Medina Allende, Córdoba, Argentina; 3 Genes digitales Bioinformatic group. Argentina; 4 Facultad de Agronomía. Universidad de Buenos Aires, Buenos Aires, Argentina; 5 Max Planck Institute for Molecular Plant Physiology, Wissenschaftspark Golm, Am Mühlenberg 1, Potsdam-Golm, D-14476, Germany

## Abstract

**Background:**

The economic importance of *Solanaceae *plant species is well documented and tomato has become a model for functional genomics studies. In plants, important processes are regulated by microRNAs (miRNA).

**Description:**

We describe here a data base integrating genetic map positions of miRNA-targeted genes, their expression profiles and their relations with quantitative fruit metabolic loci and yield associated traits. miSolRNA provides a metadata source to facilitate the construction of hypothesis aimed at defining physiological modes of action of regulatory process underlying the metabolism of the tomato fruit.

**Conclusions:**

The MiSolRNA database allows the simple extraction of metadata for the proposal of new hypothesis concerning possible roles of miRNAs in the regulation of tomato fruit metabolism. It permits i) to map miRNAs and their predicted target sites both on expressed (SGN-UNIGENES) and newly annotated sequences (BAC sequences released), ii) to co-locate any predicted miRNA-target interaction with metabolic QTL found in tomato fruits, iii) to retrieve expression data of target genes in tomato fruit along their developmental period and iv) to design further experiments for unresolved questions in complex trait biology based on the use of genetic materials that have been proven to be a useful tools for map-based cloning experiments in Solanaceae plant species.

## Background

The sequencing and annotation of genomes of various organisms alongside the deposition of the resultant information in public domain repositories has lead to the availability of vast data sets. When these data sets are compared with data coming from post-genomic experimentation they can subsequently be exploited in integrative genomics approaches. This is particularly true in plant biology, since a considerable amount of information is now available allowing the linkage of traits to either genomic DNA sequences, ESTs or proteins for a wide range of different plant species (see for example *Arabidopsis*, [1]; *Solanaceae *[2]; Grasses,[3]; Legumes, [4]). At the same time experimental data on the regulation of metabolic pathways at the whole genome level has been recently released for a handful of plant species (*Arabidopsis*, [5]; tomato, [6]; legumes, [7] and barley [8]). In the case of tomato (*Solanum lycopersicum*), Schauer et al., [9] identified 889 fruit quantitative metabolic loci (QML) and 326 yield-associated loci (YAL) distributed across the tomato genome and studied the hereditability of the fruit metabolome [10]. These combined quantitative trait loci (QTL) were identified using the *Solanum pennelli *introgression line (ILs) population [11], that has previously been utilized by several groups to identify a total of more than 2000 QTL [12]. More recently, we focused on a subset of 126 of these QTL and were able to identify a total of 88 metabolism-associated and 39 non-metabolism associated (transport, signaling, protein processing or degradation and DNA/RNA/protein-metabolism) candidate genes co-localizing with these QTL [13]. Moreover, an important observation made from these combined reports is that a large proportion of the QTL were associated with changes in whole plant morphology [9,10]. However, although these experiments provide strong clues towards elucidating the interactions between genetic, expressional and protein quality aspects underlying developmental shifts during fruit ripening, the exact mechanisms underlying these traits are, as yet, far from clear.

Recent studies have demonstrated that both pattern formation and metabolism in plants involves regulation by microRNAs (miRNAs) of transcription factors [14] and enzyme-encoding genes [15,16]. These studies, alongside the demonstration that miRNA319 regulates tomato leaf morphology [17], suggests that this level of regulation should also be evaluated with respect to the metabolic changes observed in the introgression lines. This prompted us to search for miRNA precursors and their putative target genes in the genomic regions comprising these QTL. To integrate this information here we compiled a non-redundant database of known miRNAs [18], and screened the *Solanaceae *Unigene collection [19] and completed BAC sequences from the tomato genome sequence initiative (*Solanaceae *Genome Network: http://www.solgenomics.net), for putative target sites. Target sites found in genomic clones were annotated by using two gene prediction softwares (Augustus; [20] and GenomeThreader; [21]) and aligned against *S. lycopersicom *unigenes and *Arabidopsis thaliana *peptide sequences and finally mapped onto the respective BINs (chromosomal segments) of the IL population using the molecular markers of two genetic maps (Tomato EXPEN2000 and Tomato EXPEN1992, http://www.solgenomics.net). Moreover, the expression profiles obtained from the assessment of tomato fruit development [22] of the target genes were also integrated. The resultant database, named miSolRNA, is comprised of 16 tables storing information concerning the map positions of miRNA target genes and their expression patterns as well as map positions of genes co-localizing with the previously identified QML. Relations within the whole dataset are searchable by means of the following fields: BIN, miRNA, target and keywords. Retrieved information can be set by the user in the following fields: i) QTL, indicates those metabolites and yield associated traits showing significant variations associated to the genomic regions where a miRNA target was found; ii) target localization, indicates the genetic BIN where the target was localized; iii) hit definition, shows annotations of the Unigene and/or the predicted products for the cases of target found onto genomic regions and iv) alignment, shows the alignments between the miRNA and the target site. Data extraction and conversion was performed by use of Python scripts. The data display was built using a combination of Python, Yaro Middleware on top of Web Server Gateway Interface (WSGI; [23]), Cheetah template, JQuery and SQLite for persistence.

Meta-analyses proposed here allow the linkage of genomic data with miRNA function, gene expression and metabolite profiling data. Although the resultant computational predictions should be interpreted cautiously prior to experimental confirmation, the rapid accumulation of information concerning sRNAs [24], necessitates computational, curated, relational databases of such entities in order to facilitate the construction of hypotheses aimed at defining their physiological mode of action.

## Construction and content

**Figure 1:**
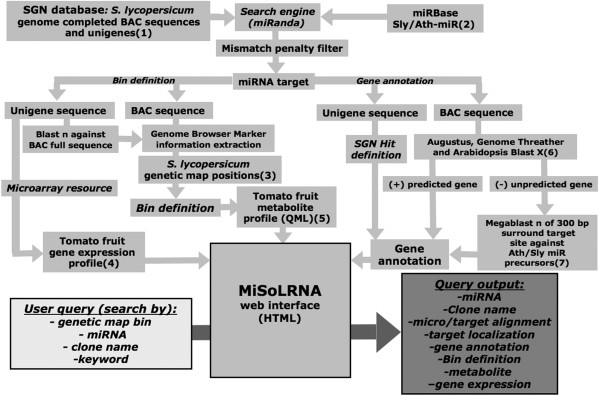
**Schematic representation of the relational pipeline used to build the miSolRNA Database**. BAC and Unigenes sequences were downloaded from the SOL Genomic Network database^(1) ^and *Solanum Lycopersicum *(Sly) and *Arabidopsis thaliana *(Ath) miRNAs from the miRBase^(2)^. Putative miRNA target sites were searched using the miRANDA software as described in the text. Recognized sequences were scored and filtered base on penalties mismatches. Both Unigenes and full length BAC sequences were positioned onto the different BINs of the *S. pennellii *IL population map[11]^(3) ^by searching all mapped markers using the on-line comparative map viewer tool described by Mueller et al, [44]. Expression data of targeted Unigenes were retrieved from a previously described microarray experiment performed along developmental and ripening processes of tomato fruit [22]^(4)^. Quantitative Metabolic Loci (QML) data previously detected by Schauer et al [9] co-localizing with targeted BIN were integrated into the relational database^(5)^. Hits were defined as the annotations of Unigenes available at the SGN data base and by *de novo *performed by Augustus, Genome Threader and Arabidopsis BLASTX annotations^(6)^. Genomic clones of Sly miR precursors were searched by BLASTN against the miRBase sequence DB as described in the text^(7)^. The entire information was incorporated into the miSOLRNA database and made available through a dedicated web interface.

The rationale of the MiSolRNA relational database is illustrated in Figure 1. The *Solanaceae *Genome Network database was searched for all completed BAC and Unigene sequences of *Solanum lycopersicum *(http://www.solgenomics.net). This sequence information was downloaded to an in-house server in order to reduce the computer time per file. In parallel, mature miRNAs sequences from two plant species (*A. thaliana *and *S. lycopersicum*) were downloaded from the miRBASE database v13.0 (http://www.mirbase.org), yielding a total of 217 miRNA entries. miRNA target site predictions using either genomic (BAC) or Unigene sequences were performed by running miRanda software with parameters set by default (http://cbio.mskcc.org/) [25]. Miranda source code was slightly modified in order to accommodate reference files with sequences larger than 100 Kbps. This was accomplished by changing these two lines: reference = (char *) calloc (100000, sizeof (char)); reference2 = (char *) calloc (100000, sizeof (char)); into reference = (char *) calloc (250000, sizeof (char)); reference2 = (char *) calloc (250000, sizeof (char)); in source code file scan.c. Outputs from this screening consisted of miRNA, BAC and Unigene labels and nucleotide target positions within a given BAC or unigene sequence as well as miRNA:target aligments. The later were analyzed and filtered based on a mismatch penalty scores assigned as follow: G:U wobble pairings = 0.5 (was not consider a mismatch), insertions/deletions (indels) = 2.0, mismatch in any position different of 2 or 7 from the 5' end of the miRNA = 1.0, mismatch in position 2-7 form the 5'end of the miRNA = 1.5. Scores values were always calculated based on 20 nt and when the query was longer, all the possible consecutive 20 nucleotides were calculated and the minimum score used. A threshold score value (<3) was used to curate results [26,27].

Those genomic regions predicted to be targeted by a miRNA were annotated automatically using the Gff3 BAC files information (containing the genome browser information) downloaded from http://www.solgenomics.net ftp site. From these sequence files, the following gene prediction information was extracted: i) gene positions predicted by the Augustus software against tomato EST, potato EST, tomato Unigene and "*de novo*" hints and ii) gene positions predicted by the Genome threader (http://www.genomethreader.org/) against tomato Unigenes supporting alignments and BLASTX alignments against the TAIR9 Arabidopsis peptides database (TAIR9_pep_20090619, located at http://www.arabidopsis.org/). Following this analysis target sites were scored as positives ("yes") or negatives ("no") when a predicted gene by any August modality was hit. Outputs obtained after the analysis of the annotation by Genome threader and those obtained by BLASTX against the Arabidopsis peptide DB are also retrievable by a single search. Moreover, when the preceding analyses did not recognize a gene, these targeted sequences were used as query in Megablast analyses for putative miRNA precursor searches against those from Arabidopsis and tomato deposited in the miRBase. The Blast parameters were -G = 3, -E = 2, -W = 20, low-complexity sequence filter and an expect value cutoff of 10^-50^.

Locations of miRNA target sites, detected within fully sequenced BACs, on the genetic map of the *Solanum pennellii *introgression lines (ILs) were determined by searching for molecular markers of both TOMATO-expen1992 and -expen2000 genetic map into the Gff3 files for each anchored BAC clone. Markers were then located to a genetic BIN at defined position ranges in each map. Unigenes predicted to be targeted by miRNAs were mapped by aligning their sequences against anchored BACs with the following BLASTn parameters: ≥90% identity and ≥95% coverage. This allowed the mapping of the putative miRNAs target sites to specific BINs of the IL map facilitating the comparison of this information with the QML and QTL previously described for fruits on these ILs by Schauer et al. [9,10]. In addition, expression data of the miRNA targets were extracted from microarray experiments performed across the developmental progression of tomato fruit ripening [22].

**Figure 2:**
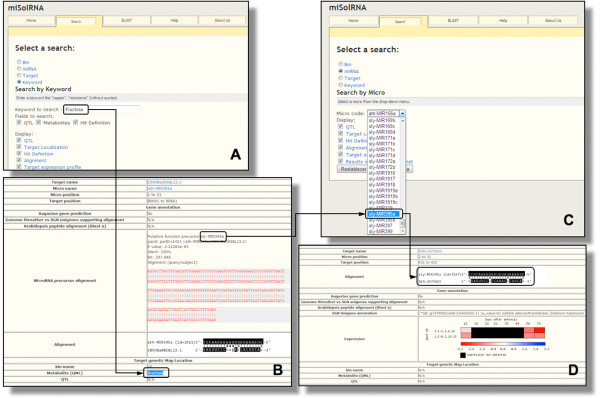
**Example of miSOLRNA interface snapshots**: (A) Fructose keyword was used in this example (A) which identified among others, the sly-MIR395a precursor co-localizing with this QML on BIN 5B of the IL genetic map (B). Searching by this miRNA in the corresponding panel (C) resulted in the identification of a target UNIGENE (SGN-U573423) annotated as a sulfate adenylyltransferase encoding gene and its expression profile along tomato fruit development and ripening (D).

The miSolRNA database is designed to display the relationship between miRNAs and their putative targets within the tomato genome. This information can be retrieved by using the following queries; genetic BIN referring to the 107 defined chromosome segments on the tomato map published by Eshed and Zamir [11], miRNA and clone (both unigenes and BAC) names. Searches can be performed by the use of displayable menus within each category for which data are available. The interface also supports querying by use of arbitrary keywords within the hit definition (referring to the publicly available genome annotation) and the QTL (referring to those previously reported by Schauer et al, [9,10]) fields. Retrievals thus show different miRNA-target relationships: genetic bin where the putative target has been mapped; annotation of the targeted hit, alignment between miRNA and its putative target sequences and, if available, the expression profile of the target gene across tomato fruit development (as published by Carrari et al, [22]). Meta-data displayed on information retrieval are linked to their corresponding source. The entire information set is given by default. However, upon users request, the different items can be called up by flagging the corresponding fields. The interface also includes a help section describing the exact definition of each search field. Figure 2 shows screenshots retrieved by the different searches available. Using the "search" panel and "Keyword" option is possible to extract information from QTL, metabolites and hit definitions fields on related miRNAs, alignments with their putative targets as well as their annotation, genebank accession, genetic position and expression profiles during tomato fruit development and ripening. Similarly, the "search by miRNA" allows retrieval of all available information for all putative target genes (annotation, genebank accession, alignment, tomato map position, co-locating QTL and expression). In all cases results can be retrieved both as HTML and Excel compatible spreadsheet formats. For bulk data manipulation the whole database is available in SQLite 3 format.

## Utility and Discussion

**Figure 3:**
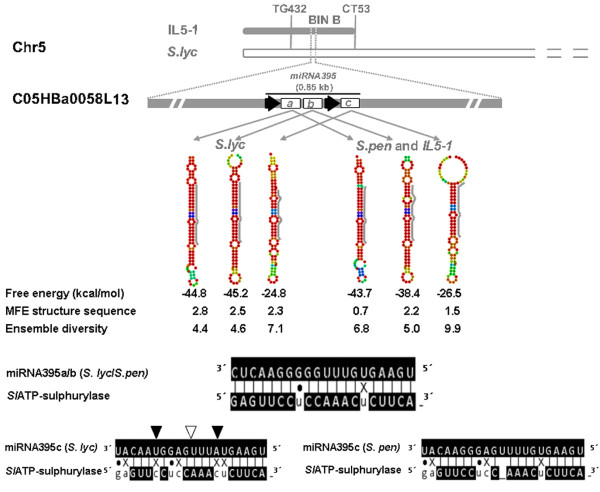
**Schematic representation of the predicted miRNA395-ATP sulphurylase mRNA interaction in an IL population (*Solanum lycopersicum *× *Solanum pennellii*)**. The top panel shows the genetic map position of miRNA395a/b/c loci in the long arm of tomato chromosome 5 (BIN B) and the physical contig of the three miRNA precursors on the BAC clone (C05HBz0058L13 GenBank Acc # AC194694). Black arrows indicate the direction of precursor's transcription and the cluster size is also indicated. The middle panel shows the predicted secondary structures of the three pre-miRNA395 (a/b/c) from the two parental lines and also from the introgressed lines 5-1. Gray lines on the right of each stem-loop indicate the regions coding the mature miRNAs. The bottom panel shows the alignments between the miRNA395a/b/c and their target gene ATP-sulphurylase mRNA. Arrows indicate polymorphism on the miRNA395c (black: substitutions and white deletion).

As pointed by Schauer et al [9,10] a large proportion of QML for several different metabolites were associated to whole plant phenotypes, suggesting that different regulatory processes at the whole plant level may be involved in the regulation of many of these QML. The biological role of miRNAs was initially thought to mainly involve the regulation of developmental patterning and cell identity [28,29]. However, the identification of additional miRNAs and their target genes suggests that miRNA functions may cover a broad range of physiological processes other than development [15,30-33]. Meta-data generated by this relational database could, therefore, potentially serve as a starting point for hypothesis generation, particularly regarding miRNA regulation exerted of tomato fruit metabolism. The analysis of the whole dataset generated here shows 7,512 possible miRNA-target interactions some of which may well be involved in the observed metabolic differences between fruit of the analyzed genotypes. Two well-known examples of miRNAs regulating plant metabolic homeostasis are miRNA395 [15] and miRNA399 (reviewed by Chiou, [34]). As a proof of concept we selected miRNA395, which was demonstrated to target ATP-sulfurylase (APS) mRNA in *Arabidopsis *cells [35] and also to be regulated itself by exogenously applied sulfate [36]. APS is a key enzyme in the first step of sulfur metabolism and its differential regulation could impact several primary pathways as demonstrated both in *Arabidopsis *roots [37] and wheat endosperm [38]. Querying the MiSolRNA database, miRNA395 retrieves several hits, among them the putative precursor of Sly-MIR395. This locus was further analyzed in details by amplifying the *S. lycopersicum *and *S. pennellii *alleles spanning a 0.8 kb fragment harboring the Sly-MIR395a, b and c precursors within the BAC C05HBa0058L13 of *Solanum lycopersicum *(GenBank Acc # AC194694) (primer sequences and PCR conditions are shown in additional file 1). Moreover, two tomato ATP-sulfurylases encoding genes (SGN-U313497 and SGN-U313496) were detected as putative targets of the Sly-MIR395. Cluster spanning the mentioned miRNA precursors was found to be physically located onto the long arm of chromosome 5 (BIN B) [11] (Figure 3), in an interval flanked by markers CT53 and TG432 (Figure 3). Furthermore, the QTL analysis reported by Schauer et al [9] showed that *S. pennellii *introgressions within this genomic region spans 19 metabolic QTL for sugars, phosphate intermediates, fatty acids, organic and amino acids contents in mature fruits as well as 5 YAL for fruit length, plant weight, Brix, harvest index and seed number per plant, respectively. To verify the database prediction we sequenced the genomic clones of the Sly-MIR395 precursors. Data showed that both *S. lycopersicum *(GB acc FJ623754) and *S. pennellii *(and also that from the IL5-1; GB acc FJ623755) alleles span a region of 852 nucleotides driven by two separated regulatory regions; one upstream of miRNA395a and -b and a second one upstream of miRNA395c immediately after the last nucleotide of the -b variant (Figure 3). These results suggest that miRNA395a and -b precursors could be transcribed as a single unit, a fact considered rare in plant systems. However, other examples of polycistronic miRNAs have been recently reported in plants [39]. *In silico *prediction of the map position of these loci was confirmed by the analysis of the sequences of three independent clones from the two parental species and the introgressed line IL5-1. This analysis additionally showed that the alleles harboured by the IL and *S. pennellii *are identical (data not shown).

Prediction of secondary structures of the pre-miRNA alleles performed by the RNAfold software (http://rna.tbi.univie.ac.at/; [40]) showed slightly different values for thermodynamic properties related to structure stability: free energy, minimum free energy (MFE) structure and ensemble diversity [41]. However, mature sequences for miRNA395a and b showed no allelic differences. This was not the case for miRNA395c which exhibited three polymorphic nucleotides including bases previously identified as being important for the miRNA-target recognition [42]. This observation thus suggests that the product of the *S. pennellii *allele may cleave the target gene mRNA more efficiently (Figure 3). The fact that the expression of ATP-sulfurylase gene is significantly down-regulated in the IL5-1 with respect to *S. lycopersicum *fruits (J. Giovannoni, personal communication) together with the allelic differences previously mentioned favor the hypothesis that the *S. pennellii *allele of miRNA395c when introgressed into the domesticated variety leads to an efficient cleavage than the *S. lycoperisum *orthologue and that these differences could be implicated in the control of a few, if not all, of the QTL mapped on this genomic region.

## Conclusions

MiSolRNA database allows the simple extraction of metadata favoring the proposal of new hypotheses about possible roles of miRNAs in the regulation of tomato fruit metabolism. It allows i) the mapping of miRNAs and their predicted target sites both on expressed (SGN-UNIGENES) and newly annotated sequences (BAC sequences released), ii) the co-location of any predicted miRNA-target interaction with metabolic QTL found in tomato fruits, iii) the retrieval of expression data of target genes in tomato fruit across development and iv) the design of further experiments aimed at addressing unresolved questions in complex trait biology. In summary, miSolRNA together with the previously released Tomato small RNAs database (http://ted.bti.cornell.edu/cgi-bin/TFGD/sRNA/home.cgi[43]), provides an insight into putative miRNA target sites within specific regions of the tomato genome and ultimately of individual genes. It also displays how these putative target genes are expressed in fruits and the co-location of these target sites with QTL for fruit metabolism. These relations provide a stepping stone for new hypotheses based on robust genetic, structural genomic, mRNA expression and metabolite profiling data.

MiSolRNA will be updated as the tomato genome sequencing project proceeds and novel sRNAs discovered. Updates will be announced in an associated RSS feed. MiSolRNA is intended as a resource to integrate information on tomato (and other *Solanaceae *plant species) metabolism and its regulation by miRNAs. Different experimental approaches already in progress in our laboratories at the Instituto de Biotecnología and at the Max-Planck-Institute of Molecular Plant Physiology will be made available through this database. Given that the in-depth analysis and understanding of metabolic regulation at the systems level will require a multidisciplinary effort, we open the database as an informative public resource for researchers focusing on experimental biology and bioinformatics. Wet experiments are under progress and they will ultimately confirm relationships suggested here such as those presented in Figure 3.

## Availability and requirements

miSOLRNA server, source code and database are freely available under the Affero GNU Public License (AGPL) at http://www.misolrna.org.

## Authors' contributions

AAB, RA participated in the design of the DB and carried out hand-curation of the DB. VG and SB developed the source code of the web application, designed the web interface and the scripts to populate the DB, MS wrote the script for sequence searching automation, MC carried out molecular cloning of genes used for proof of concept studies, ARF and SA participated in the design of the study and helped to draft the manuscript. FC coordinated the design of the DB and drafted the manuscript. All authors read and approved the final manuscript.

## Supplementary Material

Additional file 1 **Primer sequences and PCR amplification conditions **The file contains primer sequences and PCR amplification conditions used for the "proof of concept example" described in figure 3. Click here for file

## References

[B1] Rhee SY Crosby B Biological databases for plant research Plant Physiol 2005 138 1 3 10.1104/pp.104.900158 15888672 PMC1104154

[B2] Menda N Buels RM Tecle I Mueller LA A community-based annotation framework for linking Solanaceae genomes with phenomes Plant Physiol 2008 147 1788 1799 10.1104/pp.108.119560 18539779 PMC2492603

[B3] Liang C Jaiswal P Hebbard C Avraham S Buckler ES Casstevens T Hurwitz B McCouch S Ni J Pujar A Gramene: a growing plant comparative genomics resource Nucleic Acids Res 2008 36 Database D947 953 17984077 10.1093/nar/gkm968 PMC2238951

[B4] Urbanczyk-Wochniak E Sumner LW MedicCyc: a biochemical pathway database for Medicago truncatula Bioinformatics 2007 23 1418 1423 10.1093/bioinformatics/btm040 17344243

[B5] Thimm O Blasing O Gibon Y Nagel A Meyer S Kruger P Selbig J Muller LA Rhee SY Stitt M MAPMAN: a user-driven tool to display genomics data sets onto diagrams of metabolic pathways and other biological processes Plant J 2004 37 914 939 10.1111/j.1365-313X.2004.02016.x 14996223

[B6] Urbanczyk-Wochniak E Usadel B Thimm O Nunes-Nesi A Carrari F Davy M Blasing O Kowalczyk M Weicht D Polinceusz A Conversion of MapMan to allow the analysis of transcript data from Solanaceous species: effects of genetic and environmental alterations in energy metabolism in the leaf Plant Mol Biol 2006 60 773 792 10.1007/s11103-005-5772-4 16649112

[B7] Goffard N Weiller G Extending MapMan: application to legume genome arrays Bioinformatics 2006 22 2958 2959 10.1093/bioinformatics/btl517 17046975

[B8] Sreenivasulu N Usadel B Winter A Radchuk V Scholz U Stein N Weschke W Strickert M Close TJ Stitt M Barley grain maturation and germination: metabolic pathway and regulatory network commonalities and differences highlighted by new MapMan/PageMan profiling tools Plant Physiol 2008 146 1738 1758 10.1104/pp.107.111781 18281415 PMC2287347

[B9] Schauer N Semel Y Roessner U Gur A Balbo I Carrari F Pleban T Perez-Melis A Bruedigam C Kopka J Comprehensive metabolic profiling and phenotyping of interspecific introgression lines for tomato improvement Nat Biotechnol 2006 24 447 454 10.1038/nbt1192 16531992

[B10] Schauer N Semel Y Balbo I Steinfath M Repsilber D Selbig J Pleban T Zamir D Fernie AR Mode of inheritance of primary metabolic traits in tomato Plant Cell 2008 20 509 523 10.1105/tpc.107.056523 18364465 PMC2329927

[B11] Eshed Y Zamir D An introgression line population of *Lycopersicon pennellii *in the cultivated tomato enables the identification and fine mapping of yield-associated QTL Genetics 1995 141 1147 1162 8582620 10.1093/genetics/141.3.1147 PMC1206837

[B12] Lippman ZB Semel Y Zamir D An integrated view of quantitative trait variation using tomato interspecific introgression lines Curr Opin Genetics Dev 2007 17 545 552 10.1016/j.gde.2007.07.007 17723293

[B13] Bermudez L Urias U Milstein D Kamenetzky L Asis R Fernie AR Van Sluys MA Carrari F Rossi M A candidate gene survey of quantitative trait loci affecting chemical composition in tomato fruit J Exp Bot 2008 59 2875 2890 10.1093/jxb/ern146 18552354 PMC2486480

[B14] Chen K Rajewsky N The evolution of gene regulation by transcription factors and microRNAs Nat Rev Genet 2007 8 93 103 10.1038/nrg1990 17230196

[B15] Jones-Rhoades MW Bartel DP Computational identification of plant microRNAs and their targets, including a stress-induced miRNA Mol Cell 2004 14 787 799 10.1016/j.molcel.2004.05.027 15200956

[B16] Shuklaa LI Chinnusamyb V Sunkar R The role of microRNAs and other endogenous small RNAs in plant stress responses BBA-Gene Struct Expr 2008 1779 743 748 10.1016/j.bbagrm.2008.04.004 18457682

[B17] Ori N Cohen AR Etzioni A Brand A Yanai O Shleizer S Menda N Amsellem Z Efroni I Pekker I Regulation of LANCEOLATE by miR319 is required for compound-leaf development in tomato Nat Genet 2007 39 787 791 10.1038/ng2036 17486095

[B18] Griffiths-Jones S The microRNA Registry Nucleic Acids Res 2004 32 Database D109 D111 10.1093/nar/gkh023 14681370 PMC308757

[B19] Mueller LA Lankhorst RK Tanksley SD Giovannoni JJ A Snapshot of the Emerging Tomato Genome Sequence Plant Genome 2009 2 78 92 10.3835/plantgenome2008.08.0005

[B20] Stanke M Steinkamp R Waack S Morgenstern B AUGUSTUS: a web server for gene finding in eukaryotes Nucleic Acids Res 2004 32 309 312 10.1093/nar/gkh379 PMC441517 15215400

[B21] Gremme G Brendel V Sparks ME Kurtz S Engineering a software tool for gene structure prediction in higher organisms Inform Software Tech 2005 47 965 978 10.1016/j.infsof.2005.09.005

[B22] Carrari F Baxter C Usadel B Urbanczyk-Wochniak E Zanor MI Nunes-Nesi A Nikiforova V Centero D Ratzka A Pauly M Integrated analysis of metabolite and transcript levels reveals the metabolic shifts that underlie tomato fruit development and highlight regulatory aspects of metabolic network behavior Plant Physiol 2006 142 1380 1396 10.1104/pp.106.088534 17071647 PMC1676044

[B23] Eby PJ Python Web Server Gateway Interface v1.0 http://www.python.org/dev/peps/pep-0333/

[B24] Moxon S Jing R Szittya G Schwach F Rusholme Pilcher RL Moulton V Dalmay T Deep sequencing of tomato short RNAs identifies microRNAs targeting genes involved in fruit ripening Genome Res 2008 18 1602 1609 10.1101/gr.080127.108 18653800 PMC2556272

[B25] Enright AJ John B Gaul U Tuschl T Sander C Marks DS MicroRNA targets in Drosophila Genome Biol 2003 5 R1 10.1186/gb-2003-5-1-r1 14709173 PMC395733

[B26] Zhang Y miRU: an automated plant miRNA target prediction server Nucleic Acids Res 2005 33 Web Server W701 W704 10.1093/nar/gki383 15980567 PMC1160144

[B27] Alves L Niemeier S Hauenschild A Rehmsmeier M Merkle T Comprehensive prediction of novel microRNA targets in *Arabidopsis thaliana.* Nucleic Acids Res 2009 37 4010 4021 10.1093/nar/gkp272 19417064 PMC2709567

[B28] Bartel B Bartel DP MicroRNAs: at the root of plant development? Plant Physiol 2003 132 709 717 10.1104/pp.103.023630 12805599 PMC523861

[B29] Reinhart BJ Weinstein EG Rhoades MW Bartel B Bartel DP MicroRNAs in plants Gene Dev 2002 16 1616 1626 10.1101/gad.1004402 12101121 PMC186362

[B30] Adai A Johnson C Mlotshwa S Archer-Evans S Manocha V Vance V Sundaresan V Computational prediction of miRNAs in Arabidopsis thaliana Genome Res 2005 15 78 91 10.1101/gr.2908205 15632092 PMC540280

[B31] Axtell MJ Bartel DP Antiquity of microRNAs and their targets in land plants Plant Cell 2005 17 1658 1673 10.1105/tpc.105.032185 15849273 PMC1143068

[B32] Lu C Tej SS Luo S Haudenschild CD Meyers BC Green PJ Elucidation of the small RNA component of the transcriptome Science 2005 309 1567 1569 10.1126/science.1114112 16141074

[B33] Sunkar R Zhu JK Novel and stress-regulated microRNAs and other small RNAs from Arabidopsis Plant Cell 2004 16 2001 2019 10.1105/tpc.104.022830 15258262 PMC519194

[B34] Chiou TJ The role of microRNAs in sensing nutrient stress Plant Cell Environ 2007 30 323 332 10.1111/j.1365-3040.2007.01643.x 17263777

[B35] Allen E Xie Z Gustafson AM Carrington JC microRNA-directed phasing during trans-acting siRNA biogenesis in plants Cell 2005 121 207 221 10.1016/j.cell.2005.04.004 15851028

[B36] Kawashima CG Yoshimoto N Maruyama-Nakashita A Tsuchiya YN Saito K Takahashi H Dalmay T Sulphur starvation induces the expression of microRNA-395 and one of its target genes but in different cell types Plant J 2009 57 313 321 10.1111/j.1365-313X.2008.03690.x 18801012

[B37] Leustek T Sulfate Metabolism The Arabidopsis Book 2002 American Society of Plant Biologists, Rockville, MD 10.1199/tab.0017 PMC3243352 22303195

[B38] Fitzgerald MA Ugalde TD Anderson JW Sulphur nutrition affects delivery and metabolism of S in developing endosperms of wheat J Exp Bot 2001 52 1519 1526 10.1093/jexbot/52.360.1519 11457912

[B39] Boualem A Laporte P Jovanovic M Laffont C Plet J Combier JP Niebel A Crespi M Frugier F MicroRNA166 controls root and nodule development in Medicago truncatula Plant J 2008 54 876 887 10.1111/j.1365-313X.2008.03448.x 18298674

[B40] Gruber AR Lorenz R Bernhart SH Neubock R Hofacker IL The Vienna RNA website Nucleic Acids Res 2008 36 Web Server W70 74 10.1093/nar/gkn188 18424795 PMC2447809

[B41] Mathews DH Sabina J Zuker M Turner DH Expanded sequence dependence of thermodynamic parameters improves prediction of RNA secondary structure J Mol Biol 1999 288 911 940 10.1006/jmbi.1999.2700 10329189

[B42] Mallory AC Bouche N MicroRNA-directed regulation: to cleave or not to cleave Trends Plant Sci 2008 13 359 367 10.1016/j.tplants.2008.03.007 18501664

[B43] Itaya A Bundschuh R Archual AJ Joung JG Fei Z Dai X Zhao PX Tang Y Nelson RS Ding B Small RNAs in tomato fruit and leaf development BBA-Gene Struct Expr 2008 1779 99 107 10.1016/j.bbagrm.2007.09.003 18078843

[B44] Mueller LA Mills AA Skwarecki B Buels RM Menda N Tanksley SD The SGN comparative map viewer Bioinformatics 2008 24 422 423 10.1093/bioinformatics/btm597 18202028

