# Supplementary material for: miSolRNA: A tomato micro RNA relational database
Source: BMC Plant Biol. 2010 Nov 8;10:240. doi: 10.1186/1471-2229-10-240 (PMC3095322; doi:10.1186/1471-2229-10-240)
Supplement: Additional file 1 — Primer sequences and PCR amplification conditions The file contains primer sequences and PCR amplification conditions used for the "proof of concept example" described in figure 3. [file 1471-2229-10-240-S1.PDF]

Supplementary data. Primer sequences and PCR amplification conditions of the "*Proof of concept example*".

miRNA primer names	Primer sequences	BAC(*) position annealing
MiR395Tf	5´GGC ATA AGA AAT AGC AAG TGA ATC C 3´	80221 - 80246
MiR395Tr	5´CAT GTA CAC CCC TAG CGA AAA TC 3´	81256 - 81279

The miR395 amplification conditions were: first denaturation step of 94ºC, 35 cycles of 94ºC 15 seconds, 48ºC 30 seconds and 68ºC 1 minute, and a last extension step of 68ºC for 2 minutes. gDNA template was obtained as described by Hoisington et al. (1994) from leaves of *S. lycopersicum* (Heinz LA1706), *S. pennellii* (LA716) and the IL 5-2 (LA4055). 1058 pb PCR products were cloned into a pTOPO-GW vector following the manufacture instructions (Invitrogen pTOPO-GW vector cloning kit). Sequence reads from three independent clones of each genotype were generated in an ABI3101 instruments by a single reaction using the M13 forward and reverse primers. (*)BAC code: C05HBa0058L13.1. GenBank accession: AC194694.1

Hoisington D, Khairallah M, Gonzalez de Leon D. 1994. Laboratory protocols. El Baton, Mexico: CIMMYT Applied Molecular Genetics Laboratory.
